# Venous endothelial function in cardiovascular disease

**DOI:** 10.1042/BSR20220285

**Published:** 2022-11-23

**Authors:** Patrizia Dardi, Daniela Esteves Ferreira dos Reis Costa, Henrique Charlanti Reis Assunção, Luciana Venturini Rossoni

**Affiliations:** Laboratory of Vascular Physiology, Department of Physiology and Biophysics, Institute of Biomedical Sciences, University of São Paulo, Brazil

**Keywords:** Endothelium, Heart Failure, Hypertension, Venous System, Venous Tone

## Abstract

The essential role of the endothelium in vascular homeostasis is associated with the release of endothelium-dependent relaxing and contractile factors (EDRF and EDCF, respectively). Different from arteries, where these factors are widely studied, the vasoactive factors derived from the venous endothelium have been given less attention. There is evidence for a role of the nitric oxide (NO), endothelium-dependent hyperpolarization (EDH) mechanism, and cyclooxygenase (COX)-derived metabolites as EDRFs; while the EDCFs need to be better evaluated since no consensus has been reached about their identity in venous vessels. The imbalance between the synthesis, bioavailability, and/or action of EDRFs and/or EDCFs results in a pathological process known as endothelial dysfunction, which leads to reduced vasodilation and/or increased vasoconstriction. In the venous system, endothelial dysfunction is relevant since reduced venodilation may increase venous tone and decrease venous compliance, thus enhancing mean circulatory filling pressure, which maintains or modify cardiac workload contributing to the etiology of cardiovascular diseases. Interestingly, some alterations in venous function appear at the early stages (or even before) the establishment of these diseases. However, if the venous endothelium dysfunction is involved in these alterations is not yet fully understood and requires further studies. In this sense, the present study aims to review the current knowledge on venous endothelial function and dysfunction, and the general state of the venous tone in two important cardiovascular diseases of high incidence and morbimortality worldwide: hypertension and heart failure.

## Introduction

The pivotal role of the endothelium in the pathophysiology of blood vessels started with the seminal work of Furchgott and Zawadzki published in 1980 [1]. Until the late 1970s, the importance of endothelial cells for vascular biology was limited to their role as a mechanical and biological barrier between the blood and the vascular wall. However, the discovery of the obligatory role of endothelial cells in the acetylcholine-induced relaxation through the release of endothelium-derived relaxing factors (EDRF) [1] and the subsequent characterization of nitric oxide (NO) as the first EDRF [2], definitely changed the course of vascular biology. Currently, the endothelium is widely recognized as an endocrine/paracrine organ, responsible for the synthesis and release of a wide variety of factors that regulate vascular tone and growth, blood fluidity, platelet aggregation, inflammation, and angiogenesis, among other functions. Furthermore, during the past 42 years, much attention has been focused on the role of these cells in the pathophysiology of cardiovascular diseases. Despite the large number of studies involving endothelial cells, there is still much to explore about their role in venous circulation, both in health and disease. In this sense, this review article will explore studies underlying how changes in the venous tone—especially those involving endothelium-derived factors—impact the cardiovascular system in physiology and two important cardiovascular diseases of high incidence and morbimortality around the world: hypertension and heart failure.

## Venous system

The venous vessels are crucial to cardiovascular physiology and blood flow generation, as they allow the return of blood from organs and tissues back to the heart based on the difference between mean circulatory filling pressure (MCFP) and the right or left atrial pressure, which determines the venous return [3,4]. This venous return is critical for ventricular preload, cardiac workload, stroke volume, and cardiac output (CO).

Veins are considered capacitance vessels, accommodating approximately 70% of the total blood volume of a healthy individual at rest within minimal pressure changes, thus requiring more distensible and less muscular walls [5,6]. Although not all veins have the same capacitance, for example, large visceral veins are considerably more compliant than limb veins, the whole venous system allows adequate blood return to the heart, maintaining CO and organ perfusion even in critical situations, such as hemorrhage [4,7–9].

**Figure 1:**
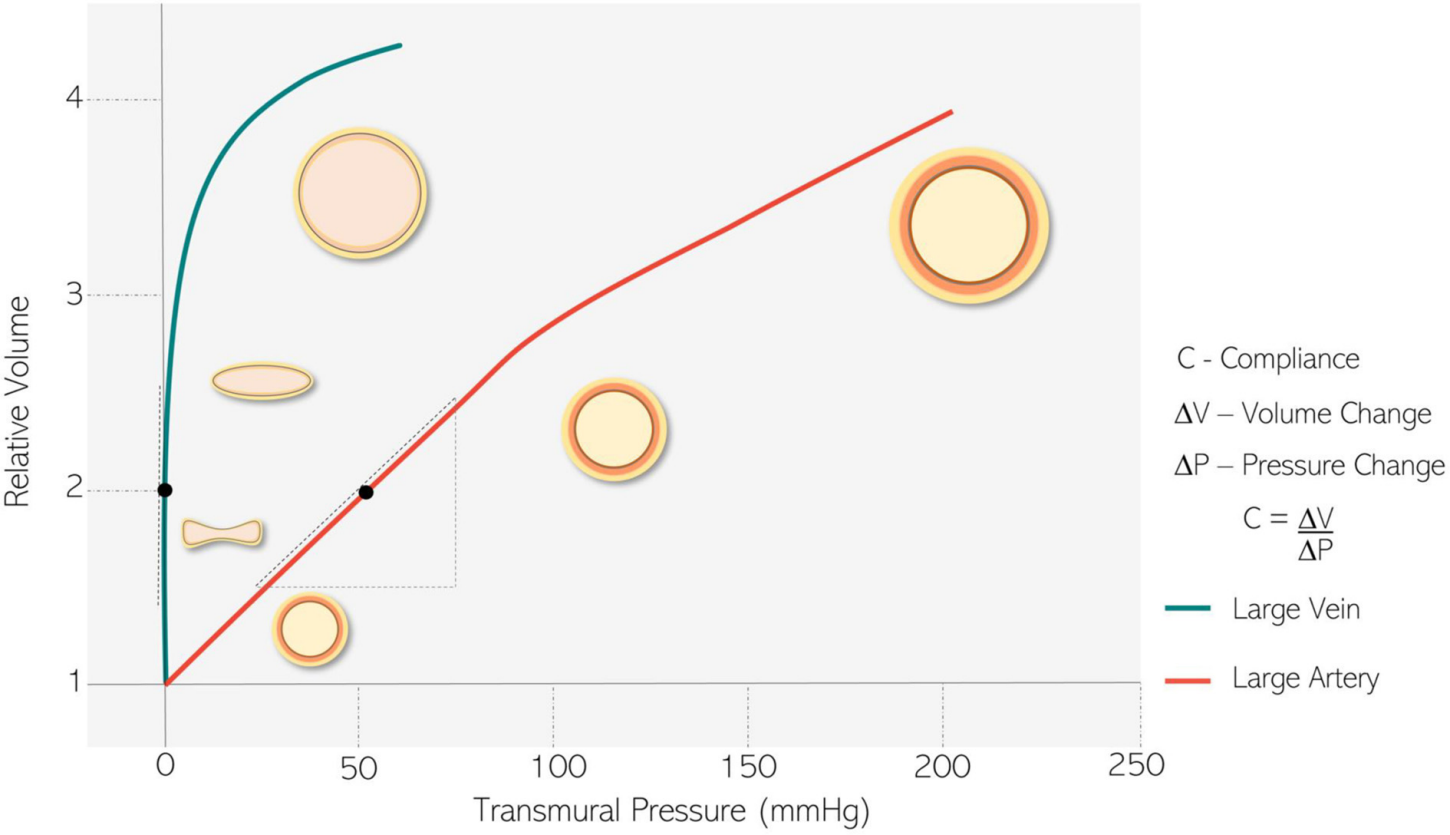
The schematic image of arterial and venous pressure–volume curve The presented graph represents the expected pressure-volume curves in large arteries (red) and veins (green). The tangent slope obtained in different values of pressure and volume is equivalent to the vessel compliance, which is characteristic of each of these vascular beds and dependent on their structural wall components. As shown, veins developed steeper slope values, once they can support higher blood volumes with small changes in pressure, which determines their greater compliance.

In general, veins have a thinner wall thickness, with less smooth muscle and elastic tissue, and more collagen, than arteries [10]. On the other hand, the adventitia layer is thicker in veins than in arteries [11,12] and, interestingly, this layer produces most of the superoxide anion (O_2_^−^) in the veins [13]. Structural differences between veins and arteries contribute to the distinct elastic behavior of these vessels. The most useful index of distensibility is compliance, which is the slope of the tangent of the pressure–volume curve (Figure 1). Thus, the steeper the slope of a pressure–volume curve, as observed in veins, the greater the compliance. Therefore, changes in the volume of veins will induce smaller changes in venous pressure compared with arteries [11]. In fact, veins are approximately 30 times more compliant than arteries [7]. However, the smooth muscle layer present in these vessels is sufficient to control venous tone (e.g., by endothelium-dependent mechanisms [14,15]), diameter, and pressure, even in small veins [12], moving blood from the periphery to the central venous compartment. Therefore, changes in venous tone by constriction or relaxation have significant effects on venous return and cardiac preload, with important adjustments in cardiac function and repercussions on arterial blood flow.

Studies comparing arterial and venous beds with similar calibers show different responses to most constrictor or dilator agonists. Although veins have a thinner smooth muscle layer and contract less in response to the high-potassium solution (KPSS) [16], angiotensin II (Ang II), endothelin-1 (ET-1), and U-46619 (a thromboxane A_2_ analog) produce higher contraction in veins than in arteries when normalized by KPSS-induced contractile response [17–20]. However, there is no consensus regarding the contractile response induced by noradrenaline in veins; increase [17,20], decrease [19], or no change [18] in noradrenaline-induced contraction have been described in veins compared with arteries. Veins also relax less in response to endothelium-dependent vasodilating agents, such as acetylcholine and ADP [20–23].

Comparisons between venous and arterial endothelium also demonstrated clear differences in the production and bioavailability of reactive oxygen species (ROS). The vena cava produces more O_2_^−^ and hydrogen peroxide (H_2_O_2_) than the aorta and has greater expression and activity of xanthine oxidase [24]. Despite this relatively greater ROS production, the vena cava also has a higher antioxidant defense associated with higher expression of CuZn-superoxide dismutase (CuZn-SOD) and catalase than the aorta [24]. Results of immunohistochemical analyzes have also revealed that xanthine oxidase, CuZn-SOD, and catalase are expressed in the endothelial layer of the vena cava [24]. In line with the production of ROS by the venous endothelium, basal O_2_^−^ production was also detected in cultured immortalized endothelial cells from the vena cava [25]. In addition, a lower ratio between reduced and oxidized glutathione (GSH:GSSG), with greater sensitivity to the inhibition of GSH production, was observed in vena cava endothelial cells than in aorta endothelial cells [26]. But despite this data, it is still unclear how these differences affect the functional aspects of the veins in contrast with the arteries.

It is well known that when the contractile or relaxing response of a blood vessel is evaluated, in addition to the intrinsic response of smooth muscle cells, there are effects resulting from vasoactive factors produced by the endothelium, perivascular adipose tissue (PVAT), and adventitia, in addition to neuro-humoral factors, that modulate these responses [27–29]. In line with this concept, endothelial cells modulate arterial and venous tone through the release of vasoactive factors that (i) act directly on vascular smooth muscle, promoting vasoconstriction or vasodilation, or even (ii) reducing or amplifying the contractile response induced by other contractile agents [1,29]. Thus, the differences observed in the contractile and relaxing responses between veins and arteries may be due to a lower/higher synthesis, bioavailability, and/or effect of EDRF and/or endothelium-derived contractile factors (EDCF). In this sense, compared with the arteries, venous endothelium produces a smaller amount of NO [30–32], and a larger amount of contractile factors, such as ET-1 [33,34] and ROS [24].

## Venous endothelium-derived factors

Studies have pointed to the participation of different EDRFs in the control of venous tone. In human veins pre-contracted or not with noradrenaline, venodilation to acetylcholine or carbachol was inhibited by a non-selective NO synthase inhibitor [14,35]. Similar results were found in rat isolated inferior vena cava and femoral veins [36–38], and in porcine pulmonary and coronary veins [31,32], where acetylcholine or bradykinin produced endothelium- and NO-dependent venodilation. These data, therefore, reveal that NO is an essential EDRF in animals and human veins. However, the specific NO synthase isoform involved in NO synthesis was not addressed in the aforementioned studies. Although both endothelial and neuronal NO synthase isoforms (eNOS and nNOS, respectively) have already been described to be expressed in pulmonary veins of rabbit [39] and vena cava, portal and pulmonary veins of rats [16,40,41], the specific role, and importance of these enzymes to the maintenance of venous tone have not been well elucidated.

Additionally, endothelium-dependent hyperpolarization (EDH) mechanisms were also shown to play a role in the inferior vena cava and femoral vein of rats, and porcine pulmonary and coronary veins [31,32,36,38]. Interestingly, the relaxing role of EDH was greater in small than large coronary veins, suggesting that, as seen in the arterial system [42,43], the contribution of EDH to the regulation of venous tone increases as vessel caliber decreases [31]. Besides NO and EDH, cyclooxygenase (COX)-derived metabolites also contribute to venodilation. Indeed, there is evidence for a contribution of COX-derived metabolites to bradykinin-induced venodilation in isolated porcine’s pulmonary and coronary veins [31,32]. On the other hand, the participation of this COX-derived vasodilator factor for endothelium-dependent relaxation was not observed in rat isolated inferior vena cava [37,38], highlighting that the contribution of COX-derived metabolite depends on the venous bed and the animal model used.

It is well known that contractile agonists also stimulate the release of EDRFs to counteract their vasoconstrictor effect. Increasing concentrations of Ang II induce contraction in isolated inferior vena cava, femoral, superior mesenteric, and portal veins of healthy rats, which is partially counterbalanced by NO since NO synthase inhibition enhances vasoconstriction induced by this peptide [44,45]. Intriguingly, in the primary culture of rat vena cava and portal vein endothelial cells, Ang II increased NO production in the vena cava, but not in the portal vein cells [41]. This indicates differences between *ex vivo* and *in vitro* studies using the same venous vessels. Endothelial cells from the primary culture of vena cava and portal vein showed, in addition to NO production, a basal production of the COX-derived metabolites, such as prostacyclin (PGI_2_) and prostaglandin F2α (PGF_2α_), a venous EDRF and EDCF, respectively [41]. However, the influence of COX-derived metabolites on Ang II-induced contraction is still not a consensus. In isolated vena cava and femoral vein, Ang II-induced venoconstriction was potentiated in the presence of a non-selective COX inhibitor [44], whereas COX metabolites did not affect the Ang II response in isolated superior mesenteric and portal veins [45]. Intriguingly, Ang II increased the production of both PGI_2_ and PGF_2α_ in the primary culture of vena cava endothelial cells, and only enhanced the PGF_2α_ production in the portal vein [41]. Once again, these results demonstrated that, in addition to NO, the release of COX-derived metabolites also counteracts the venoconstrictor effect of Ang II in a venous bed-dependent manner.

ET-1 is another potent vasoactive peptide produced and released by the endothelium [46] from both arteries and veins, although veins produce this peptide in greater amounts compared with arteries [33,34]. Moreover, the vasoconstrictor effect of ET-1 is more potent in veins than in arteries [20,34]. In the guinea pig’s mesenteric vein, venoconstriction induced by ET-1 in resting tone was partially counterbalanced by NO [47], while this effect was not observed in rats’ mesenteric vein [34]. In rat’s PGF_2α_ pre-contracted vena cava, low ET-1 concentrations induced a slight venodilation through ET_B_ receptors activation [37]. On the other hand, a higher concentration of ET-1 potentiates the venoconstrictor effect induced by PGF_2α_ through ET_A_ and ET_B_ receptor activation [37]. In addition, the ET_B_ receptor agonist, sarafotoxin 6c (S6c), also promotes venodilation that is markedly reduced (but not abolished) in the absence of the endothelium or the presence of NO synthase inhibitor, but not in the presence of non-selective COX inhibitor [37]. In contrast, in rat and guinea pig mesenteric veins, S6c caused a venoconstrictor effect under resting conditions, but this effect was counterbalanced by endothelium-derived NO and COX metabolites [34,47].

In addition to these effects, ET-1 also stimulates ROS production, H_2_O_2_ [48] and O_2_^–^ [24,49], in veins. However, in rat superior vena cava, catalase (an H_2_O_2_ scavenger) or its inhibitor did not change the venoconstricition elicited by ET-1 [48]. Furthermore, ROS scavenger tempol did not modify ET-1-induced contractile response in rat vena cava [49]; although the xanthine oxidase inhibitor reduced it [24]. Therefore, the physiological role of H_2_O_2_ and O_2_^−^ on ET-1-induced venoconstriction is still questionable.

The role of H_2_O_2_ in the veins is also intriguing. Under resting conditions, H_2_O_2_ induced concentration-dependent venoconstriction [48,50] that was abolished after COX-inhibition, and reduced after thromboxane receptor (TP) blockade [51], indicating a crucial role of vasoconstrictors metabolites derived from COX, such as thromboxane A_2_ (TxA_2_), in H_2_O_2_-induced venoconstriction. Interestingly, in PGF_2α_ pre-contracted vena cava, H_2_O_2_ induced a concentration-dependent venodilation, but this response was not seen in KPSS pre-contracted vessels, where H_2_O_2_ potentiated the venoconstriction [50]. Since the KPSS-induced contraction involves smooth muscle cell depolarization, it is possible to assume that H_2_O_2_-induced venodilation involves an EDH-mediated response.

Unlike what is seen in arteries where factors derived from the endothelium are widely studied and known, there is still no consensus on the vasoactive factors released by venous endothelium. We can assume, based on the abovementioned studies, that NO, EDH, and COX metabolites may differently contribute to venous relaxation depending on vessel location and the species studied (Table 1). On the other hand, vasoconstrictor COX metabolites and ET-1 are commonly associated with venoconstriction, although the contribution of O_2_^−^, and H_2_O_2_ to this response is less studied and still contradictory (Table 2).

**Table 1:** Summary of EDRFs identified in healthy veins, under different pre-contracted conditions and stimulus

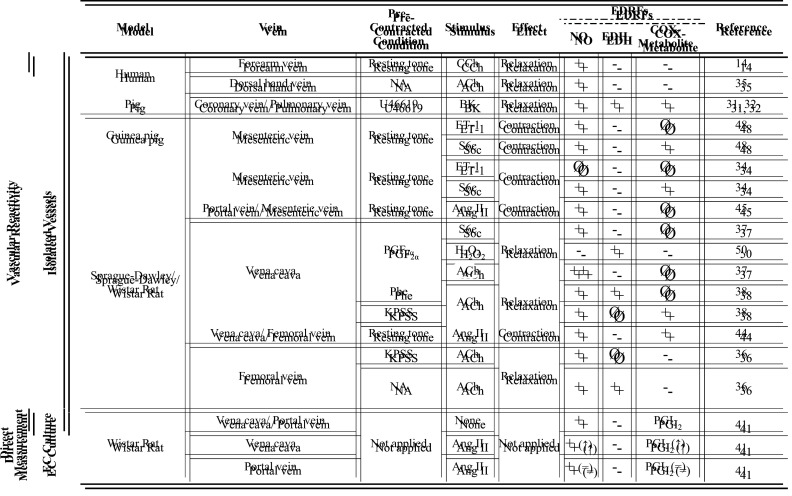

+: partial effect; ++: 100% of the effect; Ø: no effect; -: not determined; ↑: increase relative to basal unstimulated; =: no difference from basal unstimulated. Abbreviations: ACh, acetylcholine; Ang II, angiotensin II; BK, bradykinin; CCh, carbachol; EC, endothelial cell; EDH, endothelium-derived hyperpolarization; EDRF, endothelium-derived relaxing factor; ET-1, endothelin-1; H_2_O_2_, hydrogen peroxide; KPSS, high potassium salt solution; NA, noradrenaline; NO, nitric oxide; PGF_2α_, prostaglandin F2α; PGI_2_, prostacyclin; Phe, phenylephrine; S6c, sarafotoxin 6c; U46619, thromboxane A_2_ mimetic.

**Table 2:** Summary of EDCFs identified in healthy veins, under different pre-contracted conditions and stimulus

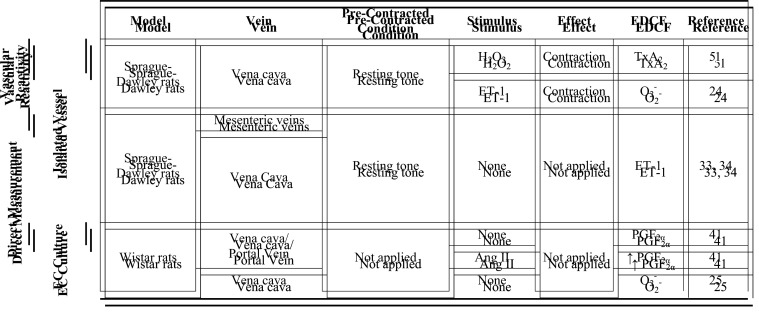

↑, increase relative to basal unstimulated. Abbreviations: Ang II, angiotensin II; EDCF, endothelium-derived contractile factor; ET-1, endothelin-1; H_2_O_2_, hydrogen peroxide; PGF_2α_, prostaglandin F2α; O_2_^−^, superoxide anion; TxA_2_, thromboxane A_2_.

The imbalance between the synthesis, bioavailability, and/or effect of EDRFs and/or EDCFs results in a pathological process known as endothelial dysfunction [29], which leads to impaired vascular tone control and contributes to the pathogenesis of cardiovascular diseases, such as hypertension [52,53], atherosclerosis [54,55], and heart failure [56,57]. However, most studies have focused on the relationship between the arterial endothelium and these diseases, with relatively little focus given to the venous system, which will be discussed in the next topics. This probably reflects the fact that most experimental *in vivo* techniques used on the arterial side of the circulation are not suitable for studies in veins, based on the morphological, anatomical, and physiological differences between these vascular beds [58]. It is also important to highlight that some of those maneuvers employed for venous tone and compliance assessment in humans—despite being used for decades—are not common for researchers who do not work with clinical trials; thus, more detailed reviews and discussions should be accessed to help understand the results and methodologies described below [9,12,58,59].

## Venous system in hypertension

Hypertension is a complex and multifactorial disorder defined as the presence of elevated systolic and/or diastolic blood pressure, which represents one of the main risk factors for cardiovascular disease and mortality worldwide [60].

Endothelial dysfunction is one important hallmark of hypertension [61–63]. In hypertensive patients, due to methodological limitations, information about regional circulations is scarce and usually limited to the forearm vasculature. Panza et al. [64] were the first to determine that patients with essential hypertension have abnormal endothelium-dependent relaxation to acetylcholine in the brachial artery from the forearm vasculature [64]. However, whether endothelial dysfunction is a cause or consequence of the hypertensive process was only determined later by Taddei et al. [65,66]. These authors found that normotensive patients genetically predisposed to developing hypertension have impaired endothelium-mediated vasodilation induced by acetylcholine, which was associated with reduced NO bioavailability, suggesting that arterial endothelial dysfunction can precede the onset of hypertension and not only be a consequence of the blood pressure increment [65,66].

Although there is extensive evidence that endothelium-dependent vasodilation is impaired in arteries of hypertensive patients, knowledge of venous endothelial function in conditions of hypertension is still quite limited. Rubira et al. [15] performed an observational study to evaluate whether hypertensive patients also exhibit venous endothelial dysfunction [15]. The authors demonstrated that hypertensive patients had a significant impairment in endothelium-dependent vasodilation induced by acetylcholine in both the dorsal hand vein and the brachial artery, while endothelium-independent vasodilation induced by NO donor remained unmodified, highlighting that endothelial dysfunction is also present in the venous system of hypertensive patients [15]. However, the mechanisms involved in venous endothelial dysfunction contributing to the pathogenesis of hypertension have not been explored.

As pointed out above, the elucidation of the mechanisms of venous function adjustment involving the participation of the endothelium and its effect on the hemodynamic profile in hypertensive patients, among other important local adjustments (e.g., coagulation, vascular permeability, etc.), is of great relevance [58]. Since the venous system contains more than half of the circulating blood, a modest decrease in venous compliance, due to reduced venodilation or increased venoconstriction, may drastically increase venous return, cardiac preload, and hence CO, triggering a vicious circle of deleterious effects on cardiac performance and adaptive remodeling of the arterial side that can lead to a sustained increase in blood pressure [10,15,67]. Accordingly, considerable evidence suggests that changes in venous vascular tone and compliance play an important role in hypertension [68–72]. However, the time course of venous changes associated with the development of high blood pressure has been examined only in a few studies.

Ferrario et al. [68] demonstrated that reduced venous compliance due to increased venous tone may be a triggering factor for renal hypertension in dogs, causing an increase in venous return and CO that preceded the increase in arterial pressure [68]. Similarly, rats subjected to a one-kidney one-clip (1K1C) renovascular hypertension showed elevated MCFP after 3 days of clipping, which continued higher up to 28 days [73]. In spontaneously hypertensive rats (SHR), MCFP was significantly elevated early during hypertension development (4–6 and 8–10 weeks of age), when the animals are still normotensive, supporting the hypothesis that the venous system has also an important contribution to the early development of hypertension [72]. Other studies have demonstrated that MCFP is elevated in SHR; however, the experiments were conducted in animals at 16–20 weeks of age, when hypertension is already established [74,75]. Taken together, these data suggest that increased venoconstriction and decreased venous compliance may be involved in both the initial and maintenance phases of hypertension.

In humans, there are relatively few *in vivo* studies evaluating venous function in hypertension. Takeshita and Mark [70] examined if venous compliance of the left forearm is altered in young men with untreated borderline hypertension [70]. The authors found that the pressure-venous volume curve was shifted toward the right in those patients, indicating reduced venous compliance compared with normotensive subjects. Additionally, the α-adrenergic blockade partially improves venous compliance in borderline hypertensive patients compared with normotensive ones [70]. In line with this study, reduced venous compliance was also found in young men with a family background of hypertension [71,76]; and once again α-adrenergic blockade was ineffective to restore this response toward normotensive patients without a history of hypertension [76].

Remarkably, reduced venous compliance observed in the initial phase of hypertension was partly related to α-adrenergic mediated venoconstriction, but may also involve other functional and/or structural mechanisms. Although structural remodeling of the vascular wall [69], neural [72,77] or humoral [78] activation of venous smooth muscle has been proposed, the nature or the main mechanism responsible for the increase in venous vascular tone and reduced venous compliance contributing to the hypertension pathogenesis remains unclear.

As well as *in vivo* experiments, it is important to highlight that only a few studies addressed *ex vivo* experiments to assess molecular mechanisms and possible signaling pathways involved in the venous system in hypertension. Among these studies, the effect of venoconstrictor factors on isolated veins was evaluated in different models of experimental hypertension. Results from these studies revealed that contractile responses to Ang II, noradrenaline, KPSS, and prostaglandin H_2_ (PGH_2_) [79,80] were increased in the isolated portal vein of SHR compared with normotensive rats, while no changes in the contractile response to Ang II were found in the portal veins from two-kidney one-clip (2K1C) renovascular hypertensive rats [79].

In another study by Morato et al. [19], it was demonstrated that isolated superior mesenteric vein from adenosine receptor blockade hypertensive rats (DPSPX-hypertensive rats) showed higher noradrenaline- and KPSS-induced contraction than normotensive rats. However, decreased Ang II-induced contraction was found in mesenteric veins from DPSPX-hypertensive rats [19]. The authors suggested that the decreased contraction of Ang II may be explained by the high levels of circulating Ang II found in this model of hypertension, which may have desensitized or down-regulated AT_1_ receptors. However, the increased response to noradrenaline may be related to an impairment of endothelial relaxation, suggesting that venous endothelial function is compromised in DPSPX-hypertensive rats [19].

The venoconstrictor effect of Ang II in hypertension was also evaluated by Loiola et al. [81] using isolated perfused mesenteric venular bed and portal vein rings of SHR. The authors found that acute administration of Ang II induced a slight but consistent constriction in isolated mesenteric venules, without significant differences between hypertensive and normotensive rats [81]. In contrast, a reduced Ang II-induced concentration-dependent response was observed in the isolated portal vein of SHR compared with normotensive rats [81]. Considering that vasoconstriction induced by Ang II is usually augmented in arteries from hypertensive animals [82–84], the authors suggested that the reduced response observed in the venous system could be related to a compensatory mechanism, avoiding an exaggerated increase in venous return to maintain CO during hypertension [81]. Interestingly, the authors also demonstrated that Ang II-induced constriction in mesenteric venules and portal veins from SHR is dependent on AT_1_ receptor activation and counterbalanced by vasodilator factors derived from COX and kinin B_2_ receptor activation, without the participation of NO in this effect [81].

Recently, Chies et al. [85] using femoral veins isolated from rats with renovascular hypertension 2K1C, reported different local mechanisms acting in a coordinated manner to mitigate Ang II responses [85]. Once again, Ang II induced very small contractile responses in femoral veins of 2K1C compared with normotensive rats. However, this lower Ang II-induced contraction was maintained in rings pre-incubated with a non-selective COX inhibitor, while the non-selective NO synthase inhibitor increased this response about three times in hypertensive vein [85]. These data suggest that NO appears to be pivotal in the modulation of Ang II responses in femoral veins of 2K1C hypertensive rats, without COX metabolites contribution [85]. Together, these findings demonstrate that similarly to what occurs in the veins of normotensive animals, a complex network of local mechanisms, mainly involving NO and COX metabolites, keeps Ang II responses under constant modulation in the venous system depending on the vein segment studied as well as the animal model of hypertension used.

As discussed earlier, in addition to Ang II, another vasoconstrictor factor determining venous tone is ET-1, which has been implicated in multiple cardiovascular functions and diseases, especially in hypertension [34,49]. Haynes et al. [86] demonstrated that in human hand veins, ET-1-induced constriction was greater in hypertensive than in normotensive patients [86]. Similarly, MCFP was higher in DOCA-salt hypertensive rats, a volume-dependent hypertensive model with elevated endothelin levels [87,88], than normotensive rats [89]. In addition, acute selective ET_A_ blockade induces a great MCFP fall only in DOCA-salt hypertensive rats [89], indicating a contribution of ET-1 in the adjustments of venous tone in hypertension.

In contrast with these findings, *ex vivo* experiments accumulate evidence indicating that venous reactivity to ET-1 via ET_A_ receptor activation is similar in the veins of DOCA-salt rats compared with normotensive rats [33]. In addition, venous pre-pro-endothelin mRNA expression did not change between groups [34]. Moreover, neither COX-derived products nor NO were involved in ET-1-induced venoconstriction in mesenteric veins from DOCA-salt rats [34]. These data suggest that mechanisms other than direct ET-1-mediated venoconstriction may explain the sustained increase in venous tone previously found in *in vivo* experiments with DOCA-salt hypertensive rats.

Interestingly, O_2_^−^ levels were increased in the venous system of DOCA-salt hypertensive rats [49,90], as well as in NO synthase inhibition (L-NNA) hypertensive rats [91], which contribute to the maintenance of the ET-1 contraction observed in isolated veins. This oxidative stress in the vena cava of DOCA-salt rats was associated with ET-1/ET_A_ receptor/NADPH oxidase activation inducing venous O_2_^−^ production [49]. Reinforcing these data, superoxide dismutase mimics tempol attenuated ET-1-induced venoconstriction only in DOCA-salt vein, suggesting the pivotal role of O_2_^−^ in venoconstriction induced by ET-1 in hypertensive rats.

Taken together, whether the abnormalities found in the venous system over the time course of primary or secondary forms of hypertension are likely a consequence of endothelial dysfunction, which impairs venodilation or increases venoconstriction, and therefore amplifies venous tone, has not yet been resolved and requires further investigations. Nevertheless, venous endothelial dysfunction can exacerbate the existing vascular damage and establish a vicious circle that contributes to the consequences of systemic hypertension [64]. Finally, we can conclude that further studies are needed to confirm whether changes in the venous system can have the same functional and structural participation, as observed in the arterial system, representing an important role for the veins during hypertension disease.

## Venous system in heart failure

Heart failure (HF) is considered a complex syndrome, with a wide range of symptoms, in which ventricular function is compromised, resulting in inadequate accommodation of venous return and deficient CO [92]. This syndrome is the converging point of several preexisting cardiac diseases and/or conditions that in the chronic stage led to systolic and/or diastolic cardiac dysfunction. Thus, this syndrome has several etiologies, with hypertension, diabetes, and ischemic heart disease being the most frequent [92,93].

Further increasing complexity, HF also comprises several clinical conditions that require the classification of the established ventricular dysfunction and the consequent physical disability of the patient, which is used to determine treatment and prognosis, or entry into clinical trials and research [92,93]. Thus, ventricular dysfunction can be categorized by its ejection fraction (EF), dividing patients into those: (i) with preserved EF (>50%; HFpEF) and (ii) with reduced EF (<40%; HFrEF)—a functional feature applied as a phenotypic marker of the HF pathophysiological mechanisms [93]. Additionally, according to the New York Heart Association (NYHA) classification system, patients are divided based on worsening disease progression to NYHA class I to IV—the former with patients without physical limitations or symptoms, and the latter with patients that are unable to perform any physical activity and that shows symptoms at rest [92].

With all the complexity and heterogeneity of this cardiovascular syndrome, researchers were already suggesting interventions to regulate vascular tone as a treatment for HF in the nineteenth century, as quickly reviewed by Cleland and Oakley [94]. However, it was not until many years later that increased systemic vasoconstriction and/or reduced vasodilation, both impacting the smooth muscle tone and blood flow distribution, were considered important hallmarks in HF [56,94–96].

In a study of venous tone *in vivo*, Litter, Wilkins, and Wood [97] studied patients with symptoms of congestive HF and measured peripheral venous distensibility with forearm volume plethysmograph [97]. The peripheral venous system of HF patients was less distensible (showing venoconstriction), when compared with hospitalized individuals with no cardiovascular diseases [97]. Additionally, a small sample of HF patients (four patients) was analyzed after clinical treatment, suggesting that the venoconstriction found would be reversed with the compensation of congestive HF and that the tone of the peripheral veins could be used as an indicator of HF prognosis [97]. In accordance with the present study, Zelis [98] evaluated the calf venous volume of HF patients (NYHA III-IV) by strain gauge plethysmography, confirming a local venous tone elevation in HF [98]. This venoconstriction was partially restored by α-adrenergic blockade and by administration of a NO donor, with both maneuvers causing venous volume expansion in HF patients, despite the NO donor not being able to reach values obtained in the control group [98]. In such manner, those responses suggested that circulating catecholamines and/or compromised smooth muscle vasodilation may contribute to the higher venous tone in HF [98].

Ikenouchi et al. demonstrated in 1991 [99], that forearm venous stiffness and venous compliance were related to the severity of congestive HF expressed by the NYHA classification system and to hemodynamic parameters (such as pulmonary vascular resistance and vascular pressure), in addition to being influenced by serum noradrenaline concentration [99]. The present study, despite being superficial—without elucidation of cellular mechanisms and signaling pathways—describes the neglected relationship between venous alterations and the development and progression of HF.

It is important to emphasize that peripheral edema observed in HF patients and the plethysmograph techniques employed in those studies are often considered possible bias factors, preventing data from being generalized to other venous beds. Thus, studies with a well-established HF rat model were developed, 3–5 weeks after left coronary artery occlusion, allowing MCFP measurements in conscious animals. Gay et al. [100] were the first to show that in rats with a left ventricle infarct size of 46%, MCFP was increased when compared with control rats; whilst effective vascular compliance was decreased, with no changes in unstressed volume [100] (considered the volume of blood remaining in the circulation when the MCFP is zero, not causing changes in transmural pressure [5,101]). This study showed that enhanced systemic venous tone is present in HF rats post-myocardial infarction, with a left ventricular end-diastolic pressure (LVEDP) higher than 15 mmHg, which would be a compensatory mechanism to maintain forward flow in the presence of decreased left ventricle function [100]. However, the mechanisms involved in changes in venous tone have not been elucidated.

Later, the same group demonstrated that myocardial infarction is associated with progressive enhancement in LVEDP, MCFP, unstressed volume, and blood volume; while venous compliance is decreased, influencing HF prognosis [102]. Also, treatment of infarcted animals with angiotensin-converting enzyme (ACE) inhibitor captopril reverted all those parameters in the HF group, reducing LVEDP, MCFP, unstressed, and blood volume, in addition to augmenting venous compliance in 32%—effects not demonstrated by hydralazine treatment [102]. Thus, the authors considered the participation of the renin–angiotensin system (RAS) in the progression of HF, suggesting that its inhibition not only promotes vasodilation on arterial beds but also in the venous system, redistributing blood volume in venous reservoirs with higher capacitance and therefore, influencing the ventricular function [102]. Further publications also reported the influence of vasopressin and Ang II receptor antagonism in the regulation of venous tone, with both treatments increasing venous compliance in HF post-myocardial infarction in rats [103,104].

To assess the mechanisms modulating venous tone, Lefer et al. [105], demonstrated that cardiac veins from a dog model of myocardial ischemia and reperfusion exhibited impaired endothelium-mediated venodilation in comparison to sham-operated dogs, despite preservation of endothelium-independent smooth muscle relaxation [105]. Since the EDRF-mediated response in cardiac veins of control animals was sensitive to non-selective inhibition of NO synthase, these authors suggested that endothelial dysfunction on this venous bed after ischemia and reperfusion is dependent on ROS production, compromising NO availability [105]. In this sense, it is possible to suggest that changes in venous endothelial function could compromise venous tone early after cardiac tissue damage, which may favor the impairment of ventricular function over time.

In accordance with this rapid loss of endothelial venodilation in cardiac bed after myocardial injury, our research group, Dardi et al. [16], assessed vena cava and thoracic aorta endothelial function of HF post-myocardial infarction rats, comparing in an *ex vivo* approach, both endothelial responses and underlying mechanisms. The results obtained showed that the acetylcholine-induced vasodilation was enhanced in the thoracic aorta, while it was reduced in the vena cava of HF rats when compared with control animals, indicating the presence of venous endothelial dysfunction in a large capacitance vein, only 4 weeks after myocardial infarction [16]. This dysfunction was the result of nNOS uncoupling (a condition where NO synthase produces O_2_^−^ instead of NO [106,107]), which contributed to the generation of ROS and oxidative stress establishment [16]. Increased ROS production and compromised catalase antioxidant defense were associated with reduced NO availability and enhanced H_2_O_2_ production in vena cava, which may collaborate to reduce venous capacitance in a large and central vein, possibly enhancing cardiac preload that could contribute to worsening left ventricular performance in HF [16].

Interestingly, a study evaluating human internal mammary artery and saphenous veins undergoing coronary artery bypass grafting, showed that H_2_O_2_ – considered an endogenous EDH mechanism in the coronary bed – induced different responses in arteries and veins [108]. While H_2_O_2_ was produced by the nNOS on the internal mammary artery, resulting in vasodilation after acetylcholine stimuli; in saphenous veins H_2_O_2_ was generated by nNOS and by COX, inducing venoconstriction [108]. With these data, the authors suggest that a possible mechanism for the saphenous veins graft loss and vasospasms after surgery may be related to H_2_O_2_ vasoconstriction in the coronary bed [108], adding further evidence of impaired venous tone after myocardial injury. Thus, these studies [16,108] reinforce the significant role of nNOS on vascular adjustments for the development and progression of cardiac diseases [57,109].

Notwithstanding, human studies are not in agreement about the EDRF/EDCF role in the regulation of venous tone in HF, with literature presenting conflicting data. Preserved flow- and carbachol-stimulated NO production [110], and atrial natriuretic peptide-induced venous tone [111], were observed in forearm veins of HF patients, while the enhanced venous response to bradykinin was observed in ACE inhibitors-treated HF patients [112]. On the other hand, Rabelo et al. [113] demonstrate a decreased vasodilatory response to acetylcholine in patients with advanced HF compared with control individuals, without differences in endothelium-independent vasodilation [113]. This compromised response to acetylcholine was significantly improved after HF clinical compensation, allowing the authors to consider the congestive HF status as an essential component in the determination of venous endothelial dysfunction [113]. Further studies are still needed to understand the real influence of HF severity on venous tone, as venous endothelial dysfunction was observed in Chagas patients with preserved left ventricle EF [114], suggesting that venous tone is compromised in cardiomyopathy disease without HF [114]. Regarding the EDCFs, HF patients present higher plasma ET-1 levels and lower constriction response in the dorsal hand vein than healthy control patients, while the venoconstriction by ET_B_ agonist S6c was similar between groups [115]. These data suggest that both ET_A_ and ET_B_ receptors mediate venoconstriction in dorsal hand veins and that HF induces a selective decrease in venous ET_A_ receptor sensitivity [115].

To summarize, few studies have evaluated the mechanisms involved in the modulation of venous tone without biasing the full complexity of HF in a living system, or in a less heterogeneous, and more controlled group of patients and rodent models. Thus, the actual condition of venous function in peripheral or central venous beds is still not well characterized in HF. Although studies with a model of HF in rodents and with humans showed endothelial arterial dysfunction, oxidative stress, and reduced NO availability associated with hemodynamic deterioration, severe cardiac dysfunction, higher hospitalization incidence, disease progression, and increased mortality risk [57,116–119]; the lack of information about the venous endothelial function and HF progression is a significant limiting factor in the pathophysiology of this syndrome, especially because of the physiological roles played by the venous bed. By neglecting the venous system in the progression of HF, we are probably missing the discovery of important key factors for the intervention of this syndrome. Therefore, the development of new studies may be essential to improve the patient's life quality and improve the prognosis of HF.

## Conclusion

**Figure 2:**
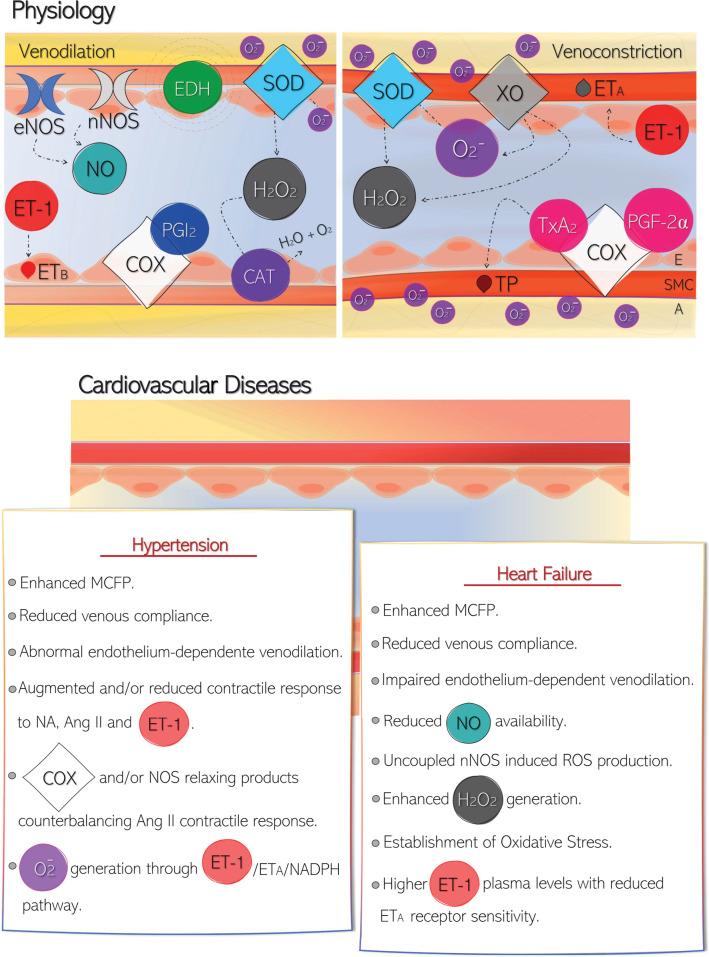
Venous endothelium-derived factors in physiology and its adjustments in cardiovascular diseases Upper panel: In physiology, the venous tone seems to be maintained through the balance of endothelium-derived relaxing factors (EDRFs) and endothelium-derived contracting factors (EDCFs). NO, EDH, COX, and PGI_2_, prostacyclin promote venodilation in most venous beds; while ET-1 (endothelin-1), O_2_^−^ (superoxide anion), and COX-derived metabolites, such as TxA_2_ (thromboxane A_2_) and PGF_2α_ (prostaglandin F_2α_), induce venoconstriction. Interestingly, hydrogen peroxide (H_2_O_2_) seems to mediate both responses, which are dependent on the vein assessed, the protocol performed, and the animal model used. Lower panel: In cardiovascular diseases, such as hypertension and heart failure, the availability of those factors that control the venous tone is poorly characterized, with few studies shedding light in the field. The main responses obtained in venous beds from patients and experimental models of these diseases are summarized in the box. Abbreviations: A, adventitia; Ang II, angiotensin II; COX, cyclooxygenase product; E, endothelium; EDH, endothelial-derived hyperpolarization; eNOS, endothelial NO synthase; ET_A_ receptor, endothelin subtype A receptor; ET_B_ receptor, endothelin subtype B receptor; MCFP, mean circulatory filling pressure; NA, noradrenaline; nNOS, neuronal NO synthase; NO, nitric oxide; PGI_2_, prostacyclin; ROS, reactive oxygen species; SMC, smooth muscle cells; TP, thromboxane receptor.

Since the discovery of the endothelium as an important regulator of vascular tone, a vast knowledge has been gathered about EDCFs and EDRFs role in the arterial system, both in physiology and in cardiovascular diseases. However, there is still a large gap regarding the role of endothelium and the release of EDCFs and EDRFs in veins. In the present review, we described evidence that the venous endothelium has an essential contribution to the physiological regulation of the venous tone, and that these cells also present considerable functional adjustments even before the establishment of some important cardiac diseases, such as hypertension and HF (Figure 2). So, although a lot of information is still missing, we can conclude that the endothelial dysfunction in the venous system can affect the venous tone, contributing to changes in venous capacitance and compliance; thus, represent a pivotal role in the development and maintenance of hypertension and HF. In this sense, new studies, especially those focusing on the mechanisms and signaling pathways involved in venous endothelium dysfunction, could be the key to avoiding and treating these diseases since the venous literature have been stagnated compared with the discoveries in the arterial endothelium in the last 42 years. Hence, there are still a lot of pieces to be elucidated in this research area, which will allow the assembly of the entire venous puzzle in physiology and cardiovascular disease.

## Abbreviations

1K1C: one-kidney one-clip
2K1C: two-kidney one-clip
ACE: angiotensin-converting enzyme
Ang II: angiotensin II
CO: cardiac output
COX: cyclooxygenase
CuZn-SOD: CuZn-superoxide dismutase
DPSPX-hypertensive rats: hypertension induced by adenosine receptor blockade
EDCF: endothelium-derived contractile factors
EDH: endothelium-derived hyperpolarizing
EDRF: endothelium-derived relaxing factors
EF: ejection fraction
eNOS: endothelial NO synthase
ET-1: endothelin-1
HF: heart failure
KPSS: high potassium salt solution
LVEDP: left ventricular end-diastolic pressure
MCFP: mean circulatory filling pressure
nNOS: neuronal NO synthase
NO: nitric oxide
NYHA: New York Heart Association
PGF2α: prostaglandin F2α
PGH2: prostaglandin H2
PGI2: prostacyclin
PVAT: perivascular adipose tissue
RAS: renin–angiotensin system
ROS: reactive oxygen species
S6c: sarafotoxin 6c
SHR: spontaneously hypertensive rats
TP: thromboxane receptor
TxA2: thromboxane A2
